# Molecular Cytogenetic Characterization of New Wheat-Rye 1R(1B) Substitution and Translocation Lines from a Chinese *Secale cereal* L. *Aigan* with Resistance to Stripe Rust

**DOI:** 10.1371/journal.pone.0163642

**Published:** 2016-09-26

**Authors:** Zhi Li, Zhenglong Ren, Feiquan Tan, Zongxiang Tang, Shulan Fu, Benju Yan, Tianheng Ren

**Affiliations:** Agronomy College, Sichuan Agricultural University, Wenjiang, Chengdu, Sichuan, 611130, China; Institute of Genetics and Developmental Biology Chinese Academy of Sciences, CHINA

## Abstract

*Secale cereale* L. has been used worldwide as a source of genes for agronomic and resistance improvement. In this study, a stable wheat-rye substitution line and 3 primary 1RS.1BL translocation lines were selected from the progeny of the crossing of the Chinese local rye Aigan variety and wheat cultivar Mianyang11. The substitution and translocation lines were identified by molecular cytogenetic analysis. PCR results, fluorescence *in situ* hybridization and acid polyacrylamide gel electrophoresis indicated that there were a pair of 1R chromosomes in the substitution line which have been named RS1200-3, and a pair of 1RS.1BL translocation chromosomes in the other 3 translocation lines, which have been named RT1163-4, RT1217-1, and RT1249. When inoculated with stripe rust isolates, these 4 lines expressed high resistance to several *Puccinia striiformis* f. sp *Tritici* pathotypes that are virulent on *Yr9*. Moreover, the different response pattern of resistance among them suggested that the diversity of resistance genes for wheat stripe rust exists in the rye. These 4 lines also showed better agronomic performances than their wheat parent. The GS indices also showed the genetic diversity of the 1RS which derived from same rye variety. The present study indicates that rye cultivars may carry untapped variations that could potentially be used for wheat improvement.

## Introduction

Common wheat (*Triticum aestivum* L.) is one of the most important crops in the world. The development of new wheat cultivars with higher yield and good resistance to diseases is the eternal goal of breeders. The alien substitutions or translocations of chromosomes between wheat and its relative species have played an important role in wheat improvement [1,2,3,4]. Many alien genes have been transferred into bread wheat through chromosome translocations from its different relative genera, such as *Secale cereale* [5,6], *Hordeum californicum* [7], *Leymus mollis* [8], *Agropyron elongatum* [9], *Haynaldia villosa* [10], *Thinopyrum* [2,11], and *Aegilops peregrina* [12].

Rye (*Secale cereale* L.) is the most valuable relative genera for the improvement of wheat genetics [13,14,15]. The 1R chromosome of rye was first introduced into common wheat from Petkus rye through a rye-wheat 1RS.1BL translocation line in Germany in the 1950s [16,17]. Many useful genes found in rye were transferred to wheat, such as genes that are resistant to against leaf rust (*Lr26*) [18], stem rust (*Sr31*) [18], stripe rust (*Yr9*) [5], and powdery mildew (*Pm8*) [5]. Moreover, the 1R chromosome also enhanced the yield potential and wide range of environmental adaptability of wheat [15,19]. Therefore, the rye 1R chromosome was used worldwide in wheat breeding programs [13]. However, the significant weakness of translocation wheat lines with the 1R chromosome derived from Petkus rye is its narrow genetic base, which is due to its single origin [5,14,15,17,20].

Stripe rust, which was caused by *Puccinia striiformis* f. sp *Tritici* (*Pst*) is usually considered to be a devastating disease in cooler areas with higher latitudes and/or altitudes [5, 21]. Since the 1990s, the *Yr9* gene from Petkus rye has not provided protection against these pathogens, due to the prevalence of virulent pathotypes [22]. For more efficient use of the 1R chromosome in wheat breeding, Ren et al. [15] put forward the idea of introducing a large amount of new genetic variation from many different rye sources into wheat. The present study reported 3 new primary 1RS.1BL translocation lines and a substitution line which were developed from the cross of the Chinese wheat cultivar Mianyang11 (MY11) and the Chinese local rye Aigan. All 4 lines showed different resistance from the translocation lines derived from Petkus rye. In this paper, we also discuss the diversity of resistant genes in the rye population. Wheat genome modification by developing more translocation or substitution lines will be valuable for the use of alien resistant genes in wheat breeding in the future.

## Materials and Methods

### Plant materials

Aigan rye (*S*. *cereale* L.) is a Chinese local rye variety which was collected from northwestern China. No specific permissions were required for Aigan rye in this study. The field studies did not involve endangered or protected species. The common wheat (*Triticum aestivum* L.) cultivar Mianyang 11 (MY11) contains the *kr1* gene and can easily be crossed with rye. Seeds of MY11 used in the present study were produced by single spike descent across several generations to create pure genetic stocks. The F1 seedlings of MY11 x Aigan were soaked in 0.05% colchicine + 3% dimethyl sulfoxide for 8 h to produce the amphidiploid (C1). The details of C1 plant production was described by Ren [23,24]. The C1 plants were backcrossed to the MY11 once or twice to produce monosomic wheat/rye addition lines. The 1R monosomic addition lines were selected and then propagated by selfing in the isolation field [23,24]. From the progeny population of 1R monosomic addition lines, the primary translocation or substitution lines were selected. In southwestern China, MY11 is highly susceptible to stripe rust. The 1RS.1BL translocation lines Chuan-nong11 (CN11), which inherited its 1RS chromosome from Petkus rye, were used as control.

### Identification of chromosomes

In this study, 5 probes were used. Three of them, Oligo-KUD15, Oligo-pSc200, and Oligo-pSc250, can be used for non-denaturing fluorescence *in situ* hybridization (ND-FISH) assays and replace the genomic DNA of rye, as a probe, to discriminate rye chromosomes in wheat backgrounds [25]. In addition, oligonucleotide probes Oligo-pSc119.2 and OligopTa535 can also be used for ND-FISH of wheat and rye [6,25,26,27]. These probes have provided an easier, faster, and more cost-effective method for the FISH analysis of wheat and hybrids derived from wheat cross with rye [25]. The preparation of the probes was described by Fu et al. [25,26]. Oligonucleotide probes were synthesized by Shanghai Invitrogen Biotechnology Co. Ltd. (Shanghai, China). These synthesized probes were diluted by using 1× TE solution (pH 7.0). *In situ* hybridization was conducted according to Fu et al. [25]. Images were captured using an epifluorescence microscope (model BX51, Olympus, Center Valley, PA, USA) equipped with a cooled charge-coupled device camera and operated with the software program HCIMAGE Live (version 2.0.1.5, Hamamatsu Corp, Sewickely, PA, USA).

### Molecular analysis

Total genomic DNA was isolated from young leaves by the surfactant cetyltrimethyl ammonium bromide (CTAB). Four sets of primer pairs were used to detect the present of 1R or 1B chromosomes. One primer pair was O11B3 (5’-ggtaccaacaacaacaaccc-3’) and O11B5 (5’-gttgctgctgaggttggttc-3’) from the *Glu-B3* gene on 1BS [28]. The second primer pair, ω-sec-P1 (5’-accttcctcatctttgtcct-3’) and ω-sec-P2 (5’-ccgatgcctataccactact-3’), was from the *Sec-1* gene on 1RS [29]. The third primer pair, TNAC1021 (5’-ctcatgcatgcgtttgttaaa-3’, 5’-ccagctgaaacaagcatcttc-3’), was specific to 1RL [30]. The fourth primer pair, PrCEN-2 (5’-aatgatcttccacgacgacg-3’, 5’-cctcgttgggaaatggtgca-3’), was designed according to nucleotides 1140–2090 of the pAWRC.1 sequence (GenBank accession No. AF245032). PrCEN-2 was used to produce a rye-specific centromeric sequence and to analyze the structure of the centromere of the 1RS.1BLchromosomes [31]. 8 SSR markers with clear bands which specific for 1RS arm were used to test the genetic diversity of 1RS chromosome arm of 4 different wheat lines (Table 1).[32] PCR was carried out in a Bio-Rad iCycler thermal cycler (Bio-Rad Laboratories, Inc., Hercules, CA, USA). DNA was amplified with 0.5 U Taq DNA polymerase enzyme, 1X buffer, 1.5 mM MgCl_2_, 200 μM dNTPs, 10 μmol primer, and 50 ng DNA in a total volume of 25 μL. After initial denaturation for 4 min at 94°C, each cycle included 30 s of denaturation at 94°C, 30 s of annealing at 60°C (for TNAC1021, the annealing temperature was 55°C), and 1 min of extension at 72°C. A final extension for 5 min at 72°C followed the 30 cycles. The products of PCR amplification were separated on 1% agarose gel. Baesd on the results of SSR, genetic similarity (GS) indices of 1RS arm among 4 lines were calculated by the software NTSYS-PC (version 2.10e) [33].

**Table 1:** The sequences of SSR markers which were specific for 1RS chromosome.

Primers name	Forward sequence	Reverse sequence
TSM 92	GGAGAGAGGATGGAGGTCGT	CAAACTTTGGCTTCGCTCTT
TSM108	GACTCATGGTCCAAGGGAGA	TTAGAGGGTCCTCCGAAACC
TSM143	CCACATCATCACCTAACTCCAG	CAGCTTTCGGAAATGGAGAC
TSM230	TTGCGGGCTCTCATATCTCT	GTGGCACAACGCTATACGG
TSM264	GGCTACAAGTTTACAGCCCACT	TCGCAATTTGTTTGTTCACC
TSM319	CCTGGAGGGAGATCCTGTTA	TGGACTAGGATGGGAGATGAA
TSM422	GTCCTGCTGCTACTGTGCTG	CACACTCGCATCCTTTGCTA
TSM469	GCTGATGGGTACTGGTTTGC	ATGTCGGTGGTCAATCCTTC

### Electrophoretic detection of ω-secalin and gliadin proteins

Electrophoretic detection of ω-secalin and gliadin proteins (A-PAGE) was conducted as described by Li et al. [6] with minor modifications. For the extraction of gliadin and ω-secalin proteins, the crushed wheat seeds were incubated in 25% (v/v) ethylene chlorohydrin with 0.05% methyl green for 12 h at room temperature with vortex mixing. The suspension was then centrifuged at 10,000g for 10 min in a microfuge. Approximately 50 μL of the supernatant was collected and used for electrophoresis. Samples were loaded onto 2-mm-thick 10% acrylamide gels and were buffered with 0.5% (w/v) N’N-methylenebisacrylamide at pH 3.1. The proteins were fractionated at a constant voltage of 500 V for approximately 180 min until the tracking dye ran off the gel. The gels were stained in 10% trichloroacetic acid (TCA) with 0.04% Coomassie Brilliant Blue G-250 and destained in 12% TCA.

### Resistance analysis

The substitution and translocation lines and their parents were examined for resistance to stripe rust in the greenhouse as described [34]. The *Pst* pathotypes CYR31, CYR32, CYR33, and a new emerging isolate SY5, which is virulent in the field to many newly released wheat cultivars, were used to inoculate the wheat plants. The *Pst* pathotypes and isolates were provided by the Plant Protection Institute, Gansu Academy of Agricultural Sciences, China. Infection types (IT) are scored based on the 0–9 scale, as described by Wan et al. [34]. IT 0–3 are considered resistant, IT 4–6 are intermediate, and IT 7–9 are susceptible. Wheat cultivar CN11, which inherited its 1RS.1BL translocation chromosome from the Russian wheat cultivar Aurora, was used as the control.

### Field experiments for determining yield components

Plants were grown in Qionglai District at Chengdu Plains, China, in 2015 following standard cultivation practices and under irrigated conditions. Entries were arranged in a randomized, complete block design with three replications, in 3 m-long plots, each consisting of eight rows spaced 25 cm apart, at a plant density of 160 seedlings/m^2^. A 1-m length from the center row of each plot was cut at ground level before harvest to determine yield components [15]. Fungicide was applied to seedlings and at heading to control powdery mildew, stripe rust, and fusarium head blight.

## Results

### Characterization of new translocation and substitution lines

**Fig 1:**
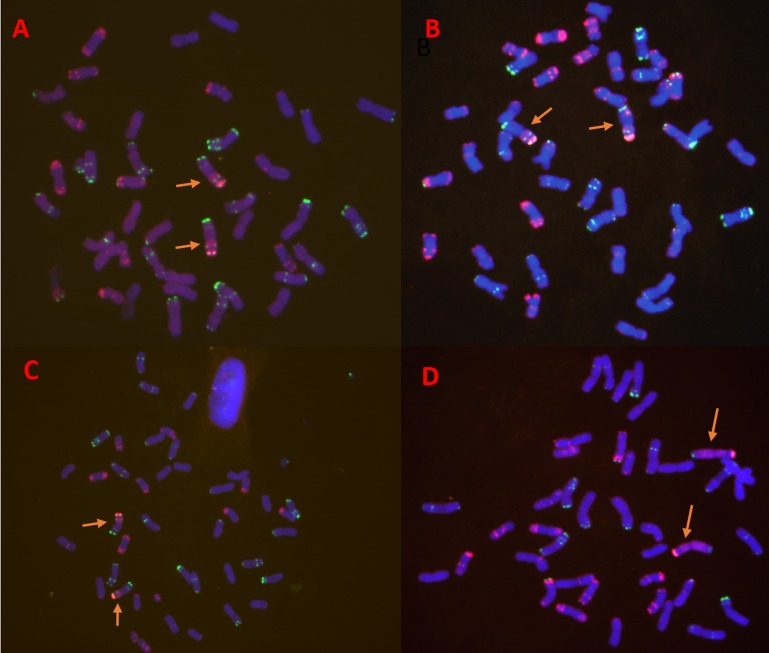
FISH of root tip chromosomes in wheat-rye translocation and substitution lines which originated from MY11 x Aigan rye. The arrows indicate wheat-rye translocated chromosomes or 1R substituted chromosomes. A. RT1249; B. RT1163-4; C. RT1217-1; D. RS1200-3.

To identify the chromosome construction of the progeny of 1R monosomic addition lines which were derived from the cross of MY11 and Aigan rye, FISH, PCR, and A-PAGE were used. Three new primary translocation lines and a substitution line were selected. Seeds used in this study were harvested from 3 generations. The results of FISH (Fig 1) showed that RT1163-4, RT1217-1, and RT1249 (2n = 42) contained a pair of wheat-rye 1RS.1BL translocation chromosomes, RS1200-3 (2n = 42) contained a pair of rye 1R chromosomes, and the 1B chromosomes of wheat were absent. Thus, the results of the cytogenetic analysis indicated that these 4 lines were cytogenetically stable, containing a pair of 1BL.1RS translocation chromosomes and a pair of 1R (1B) substitution chromosomes, respectively.

**Fig 2:**
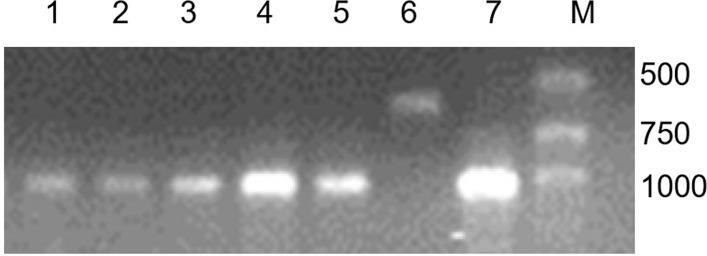
PCR results of 4 specific primers: O11B3 and O11B5, ω-sec-P1 and ω-sec-P2. Lane 1 = RT1249; lane 2 = RT1163-4; lane 3 = RT1217-11; lane 4 = RS1200-3; lane 5 = CN11; lane 6 = MY11; lane 7 = Aigan rye; lane M = marker.

Molecular markers were also used for identify these wheat translocation/substitution lines. Primer pairs O11B3 and O11B5 are the specific primers of 1BS, and a 630 bp fragment would be amplified by them. Another primer pair, ω-sec-P1 and ω-sec-P2, are the specific primers of 1RS, and they can amplified a 1076 bp fragment. The PCR results showed that only wheat cultivar MY11 amplified a 630 bp band. Both the translocation lines and the substitution line can amplify a 1076 bp band but no 630 bp band was amplified (Fig 2).

**Fig 3:**
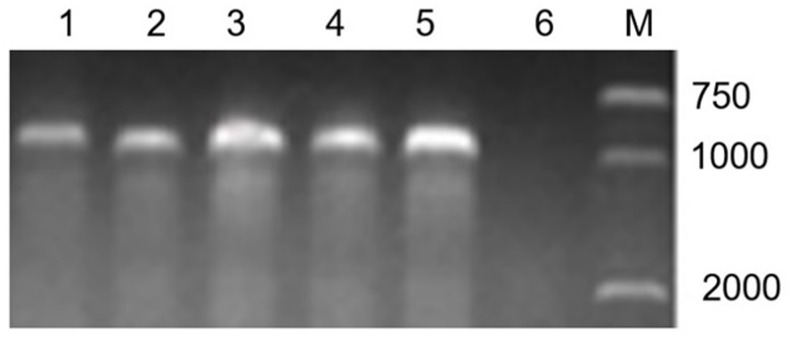
PCR result of primer PrCEN-2. Lane 1 = RT1249; lane 2 = RT1217-1; lane 3 = RT1163-4; lane 4 = RS1200-3; lane 5 = Aigan rye; lane 6 = MY11; lane M = marker.

The primer PrCEN-2 can amplify a fragment of about 1000 bp of rye centromere repetitive sequence, and the results showed that all lines amplified a band with the expect size, and no product was amplified from the DNA of MY11. It was indicated that not only the substitution line, but also the translocation lines contained the full 1RS arm of rye (Fig 3).

**Fig 4:**
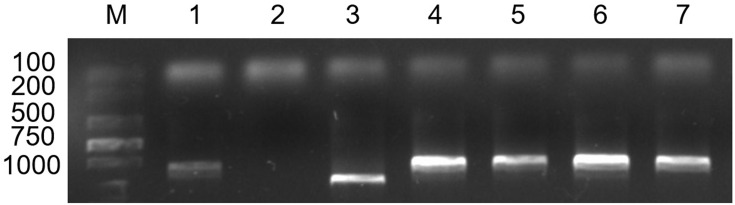
PCR results of primer TNAC1021. Lane 1 = MY11; lane 2 = water; lane 3 = RS1200-3; lane 4 = RT1249; lane 5 = RT1217-1; lane 6 = RT1163-4; lane 7 = CN11; lane M = marker.

The primer TNAC1021 can amplify a specific fragment from the rye 1RL chromosome arm. This primer could distinguish the 1RL chromosome under the wheat genome background [30]. The results showed that only the substitution line RS1200-3 can amplify the expected band (Fig 4). It was indicated that RS1200-3 contained a pair of 1R chromosomes, which was a 1R (1B) substitution line.

**Fig 5:**
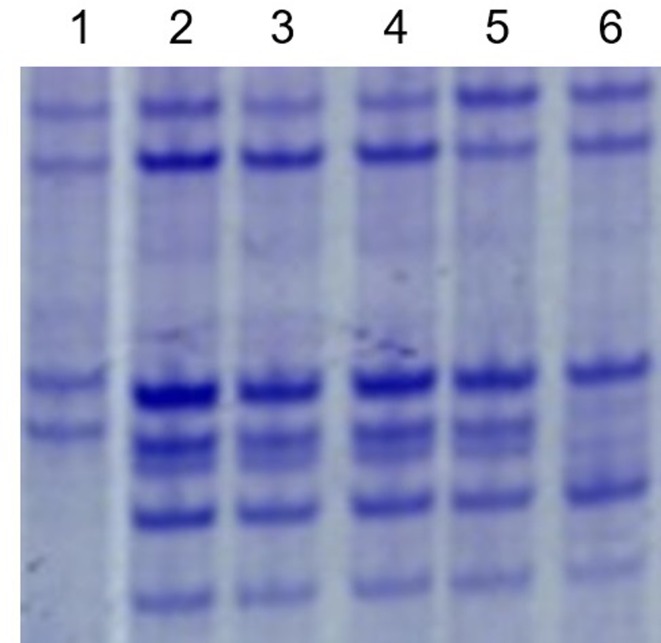
A-PAGE separations of ω-secalins and gliadins from primary 1RS.1BL translocation lines and the substitution line. Lane 1 = wheat parent MY11; lane 2 = RT1249; lane 3 = RT1217-1; lane 4 = RT1163-4; lane 5 = RS1200-3; lane 6 = CN11.

The expression of the genes at the Sec-1 locus in the 1RS chromosome arm was investigated by A-PAGE. All lines exhibited normal expression for the genes at the Sec-1 locus (Fig 5). It was also indicated that all translocation and substitution lines contained the 1RS (1R) chromosome.

### Analysis for resistance to stripe rust

Wheat parent MY11 was highly susceptible to the 4 *Pst* pathotypes, while Aigan rye was highly resistant (Table 2). Wheat cultivars CN11, whose 1BL.1RS chromosomes came from the Russian wheat cultivar Aurora (*Yr9*), were also highly susceptible to four *Pst* pathotypes (Table 2). The translocation lines and substitution line exhibited better resistance to stripe rust than their wheat parent. Although these 4 lines were derived from the same parents, they showed different resistance patterns. The translocation line RT1249 was highly resistant to CYR32 and SY5, but it was susceptible to CYR31 and CYR33. The translocation line RT1163-4 was highly resistant to CYR31 and SY5, but it was susceptible to CYR32 and CYR33. The translocation line RT1217-1 was highly resistant to all pathotypes except SY5. The substitution line RS1200-3 was highly resistant to CYR32 and CYR33 but was found to be intermediate to CYR31 and susceptible to SY5 (Table 2).

**Table 2:** Resistant analysis of translocation/substitution lines and their wheat parent to stripe rust when inoculated with epidemic pathotypes and isolates of *Puccinia striiformis* f. sp. *Tritici*, *(Pst)*.

Materials	*Pst* pathotypes and isolates			
	CYR31	CYR32	CYR33	SY5
RT1249	7	0	7	0
RT1163-4	0	7	6	0
RT1217-1	0	0	0	6
RS1200-3	4	0	0	7
Aigan rye	0	0	0	0
MY11	9	9	9	9
CN11 (control)	8	8	8	8

### Effect of chromosome translocation or substitution on agronomic traits of wheat

Significant differences (P < 0.05) between the translocation/substitution lines and their wheat parent were observed. Compared with their wheat parent MY11, RT1163-4 showed significantly reduces of plant height (PH), and displayed significantly increased of spikelet number per spike (SLN), kernel number per spike (KN) and grain yield (GY); RT1249-3 showed significantly reduced of KN; RT1217-1 showed significantly increased of number of spikes per square meter (NS); The substitution line RS1200-3, showed significantly reduced of 1,000-kernel weight (TKW), kernel weight per spike (KW), harvest index (HI), PH and GY, but displayed significantly increased of SLN, KN and NS (Table 3).

**Table 3:** Comparisons of agronomic traits among substitution, translocation lines and their wheat parent.

lines	PH (cm)	SLN(per spike)	KN(per spike)	KW(per spike)	TKW (g)	NS (m^-2^)	GY(kg/ha)	HI(%)
**RT1249-3**	104.91a	20.61b	37.97c	1.98b	52.14ab	297.3ab	5886.5b	43.56a
**RT1163-4**	84.63c	22.21a	48.84a	2.47a	52.42ab	296.5ab	7329.6a	45.47a
**RT1217-1**	94.20b	20.53b	42.88b	2.17ab	49.10b	311.7a	6764.9ab	44.39a
**RS1200-3**	61.60d	22.39a	49.86a	1.37c	27.19c	324.4a	4445.3c	38.89b
**Mianyang 11(wheat)**	98.06ab	20.24b	43.86b	2.19ab	47.86b	263.8b	5774.2b	43.74a
**Chuan-Nong11 (CK)**	88.74bc	22.13a	45.12ab	2.48a	57.19a	266.2b	6605.2ab	43.67a

Values with the same letter in the same column do not differ significantly at P<0.05. plant height (PH), spikelet number per spike (SLN), kernel number per spike (KN), kernel weight per spike (KW), 1,000-kernel weight (TKW), number of spikes per square meter (NS), grain yield (GY), and harvest index (HI).

### Genetic diversity of 1RS chromosome among 4 different lines

8 SSR primers (Table 1) were utilized to analyze genetic diversity among various 1RS chromosomes. Analyses revealed highly polymorphic patterns for different 1RS chromosomes when using these primer pairs. For 4 lines, a total of 93 amplified bands were scored from reactions with the 8 primers. All the primers could amplify bands polymorphic except primer TSM108. These results indicate a high level of microsatellite polymorphisms in the 1RS chromosome arm in 4 translocation/ substitution lines when examined using these SSR markers.

GS indices for the 4 1RS chromosomes of translocation/substitution lines were calculated based on the patterns of bands amplified with the 8 SSR primers. GS indices varied broadly from 0.4444 to 0.8056. The highest GS index (0.8056) among the 1RS chromosomes was observed between the tranlocation lines RT1217-1 and RT1163-4, whereas the lowest GS index (0.4444) was found between the translocation line RT1163-4 and RT1249, as well as the substitution line RS1200-3 (Table 4). These results demonstrate great genetic diversity within 1RS chromosome arms which were all derived from Aigan rye.

**Table 4:** Comparison of genetic similarity (GS) indices of 1RS chromosome among 4 wheat lines based on 8 SSR analysis.

	RT1163-4	RT1217-2	RT1249	RS1200-3
RT1163-4	1.0000			
RT1217-1	0.8056	1.0000		
RT1249	0.4444	0.4722	1.0000	
RS1200-3	0.4444	0.5278	0.7222	1.0000

## Discussion

### Development of new wheat-rye translocation and substitution lines

Translocation and substitution lines are important materials in the study of genetics, physiology, and phytopathology, and also are important genetics resources for wheat breeding [1,2,5,35,36,37]. Development a primary translocation or substitution lines is not easy [15]. Ren et al. [23,24] discovered that wheat chromosome pairing was disordered in the monosonic addition lines due to the presence of a single-rye chromosome. High frequency of breakage and fusion between rye and wheat chromosomes occurred during meiosis, then translocations or substitutions were able to select from the offspring. In the present study, 3 primary 1RS.1BL translocation lines and a substitution line were developed and selected from the offspring of the cross of rye variety Aigan and wheat cultivar MY11. Each line was derived from an independent offspring population of a single amphidiploid plant and their common wheat parent, a pure line isolated from cultivar MY11. However, since these rye varieties are outcrossing populations, the 1R chromosomes of these lines were presumed to be genetically variable [15,33,38,39].

### Identification of new wheat-rye translocation and substitution lines

Since large populations need to be screened to obtain translocations or substitutions, a more reliable and easier means of identifying the alien chromatin is needed. A-PAGE can identify the expression of the Sec-1 locus of chromosome 1R (1RS) [6]. Although A-PAGE is efficient for screening the 1R chromosome in large number of unknown wheat lines, it can only identified the present of 1RS chromosome in wheat genome background. A-PAGE cannot distinguish the events of translocation, substitution, or addition. It also cannot distinguish the homozygote and heterozygote. Genomic *in situ* hybridization (GISH) is a powerful technique for visualizing alien chromatin in wheat-alien hybrids. However, GISH cannot distinguish which alien chromosomes were transferred to the wheat genome. In the present study, FISH, using oligonucleotide as probes, showed that a pair of rye 1R chromosomes or a pair of 1RS.1BL translocation chromosomes was transferred in the wheat genome. These oligonucleotide probes have provided an easier, faster, and more cost-effective method for the FISH analysis of wheat [25]. Moreover, the use of molecular markers was another efficiency way to identify alien chromatin [30,40,41]. Many molecular markers have been developed and are specific for each rye or wheat chromosome, or specific for an individual locus of rye or wheat, or specific for a rye or wheat genome [29,30,40,42,43]. The use of molecular analysis can increase the efficiency of detecting alien chromatin introgressions [44]. In this study, 4 pairs of markers were used each of which were specific to 1RS, 1BS, 1RL and the centromere of rye. The PCR results of the specific primers proved the presence of 1R (1RS) and the absence of 1B (1BS). The rye centromere-specific primer PrCEN-2 was used to analyze the construction of the centromere of the translocation lines, which indicated that the 1RS chromosome arm was joint with the 1BL chromosome arm at the centromere region.

### The diversity of resistance to stripe rust of rye 1R chromosome

The 1RS.1BL translocation from the Russian wheat cultivar Aurora or Kavkaz, in which the 1RS arm was derived from Petkus rye, has been the most widespread alien translocation in wheat breeding [14,15,45,46]. Hundreds of commercial cultivars containing this 1RS arm have been released [13]. Many researchers have developed new 1RS.1BL translocation cultivars by crossing different common wheat cultivars with several existing 1RS.1BL translocation lines. Therefore, the sources of the 1RS chromosome arm were limited, and there are very few genetic variations in this 1RS arm [14,15]. Thus far, only a few other sources of 1RS have been introduced into the wheat genome [5,15,31,19,47,48,49,50]. Of these translocation lines, however, far fewer have reached significant commercial production [5]. Not every translocation or substitution lines could be used as material in a wheat breeding program.

Since the 1980s, the resistant genes *Yr9*, *Pm*8, *Lr26*, and *Sr31*, located on the 1R chromosome, have lost resistance against new respective pathogens [51]. Therefore, the frequency of rye 1R chromosome in recently developed wheat lines has declined. However, rye is a cross-pollinated plant, and the population of a variety is often genetically heterozygous [33,39]. The resistance ability to stripe rust was significantly different among the wheat lines from which 1R chromosomes were derived from different rye sources [5,15,31]. It was suggested that numerous variations can be expected in rye varieties.

In the present study, 3 new primary 1RS.1BL translocation lines and a new 1R (1B) substitution line, whose 1R chromosomes were derived from same Chinese rye variety Aigan, exhibited different phenotypes for resistance to infection by 4 *Pst* pathotypes. All 4 lines showed better resistance to the wheat cultivar CN11 with the gene *Yr9* (Table 1). Because the wheat parent MY11 was highly susceptible to all of the *Pst* pathotypes, it indicates that the resistant gene(s) in these lines must be located on these 1R chromosomes. The interesting thing is that, although the 1R (1RS) chromosome of these 4 lines came from the same rye variety Aigan, they showed different responses to inoculated *Pst* pathotypes in the greenhouse. The rye chromatins of these 4 lines were coming from the same rye variety. But they did not come from the same pollen. It suggested that there is high sense of genetic diversity in rye varieties, even in a same rye population [33]. In the present study, because these translocation and substitution lines were derived from same parent plant, their obvious difference in resistance to stripe rust would come from different resistant gene(s). Moreover, several new resistance genes are perhaps located on the 1RL chromosome arm. In the present study, the results suggest that the diversity of genes resistant genes to wheat stripe rust exist in rye. These genes can be generated by mutations and maintained in rye populations [5,15,31], and they are an important genetic resource for wheat genome modification and wheat breeding programs. Also, these 4 1RS/1BL translocation lines, or the 1R(1B) substitution line, are good resources for wheat breeding programs. The research of the mechanism of the genetic diversity of the resistance genes is in progress.

### The genetic diversity of 1RS chromosome which were derived from Aigan rye

The 4 1RS chromosomes which were derived from Aigan rye showed highly genetic diversity (Table 4). Rye is a crosspollinated plants, the rye varieties usually are complex population with mixed genetic background. The 1RS arm in different individual rye plant usually contains several alleles of resistance genes or different resistance genes is not surprised [15,22,31,48]. Also, because of the interaction between genes on 1RS and wheat background[15], and the accompanying mutations which were happened during the course of translocation[6], it resulted more genetic diversity occurrence in 1RS.1BL translocations derived from the same wheat parent and rye variety, which could be utilized as valuable resources for wheat improvement.

### The breeding value of translocation and substitution lines

The relative effects of the 1R chromosome on agronomic characters were determined in the previous studies [15,31,48]. Translocations involving 1RS or substitutions involving 1R were considered good for agronomic performance [13,19,15,31]. Compare with wheat parent MY11, all translocation lines and substitution line exhibit more slender leaves, flexible stalks, tight plant type, and NS. Although the substitution line RS1200-3 has higher SLN and KN, but the lower KW and TKW cause the yield of RS1200-3 is significant lower than MY11. The 3 translocation lines showed genetic diversity on agronomic performance, however, all of them exhibit better agronomic characters than their wheat parent MY11. Especially the RT1163-4 exhibit lower plant height, bigger spikes, significantly higher GY, showed very good value for wheat breeding program.
